# Antiinflammatory Activity of *Piper longum* Fruit Oil

**DOI:** 10.4103/0250-474X.57300

**Published:** 2009

**Authors:** A. Kumar, S. Panghal, S. S. Mallapur, M. Kumar, Veerma Ram, B. K. Singh

**Affiliations:** M. M. C. P., M. M. University, Mullana, Ambala-133 203, India; 1 Department of Pharmaceutical Sciences, S. B. S. P. G. I., Dehradun-248 161, India; 2 Department of Pharmacy, Kumaon University, Nainital- 263136, India

**Keywords:** *Piper longum*, antiinflammatory activity, rat hind paw edema method, carrageenan

## Abstract

In the present study, antiinflammatory activity of the *Piper longum* dried fruit's oil was studied in rats using the carrageenan-induced right hind paw edema method. The activity was compared with that of standard drug ibuprofen. The dried fruit's oil inhibited carrageenan-induced rat paw edema. The results indicated that the dried fruit's oil produced significant (*p*< 0.001) antiinflammatory activity when compared with the standard and untreated control.

Prolonged uses of both steroidal and non steroidal antiinflammatory drugs are well known to be associated with peptic ulcer formation[2]. Hence, search for new antiinflammatory agents that retain therapeutic efficacy and yet are devoid of these adverse effects is justified. The enzyme, phospholipase A_2_ is known to be responsible for the formation of mediators of inflammation such as prostaglandins and leukotrienes which by attracting polymorphonuclear leucocytes to the site of inflammation would lead to tissue damage probably by the release of free radicals. Phospholipase A_2_ converts phospholipids in the cell membrane into arachidonic acid, which is highly reactive and is rapidly metabolized by cycloxygenase (prostaglandin synthase) to prostaglandins, which are major components that induce pain and inflammation[3,4].

*Piper longum* linn[1] (piperaceae) is a tropical climbing shrub widely distributed throughout India. *Piper longum* is used for the treatment of respiratory tract diseases like cough, bronchitis, asthma and cold, as counter-irritant and analgesic it is applied locally in muscular pain and inflammation and internally it is used as a carminative in conditions such as loss of appetite, sleeplessness and abstruction in liver and spleen[5]. A review of literature did not reveal any reports on the antiinflammatory activity of essential oil of *Piper longum* dried fruit. The present study is therefore an attempt to assess the efficacy of this indigenous herb for its antiinflammatory activity in rats using carrageenan-induced right hind paw edema method.

Dried fruits of *Piper longum* were obtained from the ayurvedic shop situated in the market. The dried material was powdered and oil was extracted with distilled water using Clevenger's extractor. After the separation of oil from the oil-water emulsion by using the separating funnel, it was subjected to various physical and chemical tests. After analysis (specific gravity: 0.8914, refractive index: 1.4678 and other tests for essential oil) it was found that the oil was essential oil. Rats of either sex and of approximately the same age, weighing about 100-190 g were used for the study. They were housed in polypropylene cages and fed with standard chow diet and water *ad libitum*. The animals were exposed to alternate cycle of 12 h of darkness and light each. Before each test, the animals were kept under fasting for at least 12 h. Experimental protocols have received approval of the Institutional Animal Ethics Committee (IAEC, Reg. No.273/CPCSEA).

For carrageenan-induced paw edema model[6], healthy rats (100-190 g) of either sex were grouped into four groups containing five animals per group. First group (negative control) received normal saline solution (10 ml/kg) and second group (positive control) received standard drug ibuprofen (100 mg/kg). Third and fourth groups received test drug (essential oil of *Piper longum*) with different doses 0.5 ml/kg and 1 ml/kg orally with the help of an oral catheter respectively. After 1 h, the rats were challenged with subcutaneous injection of 0.1 ml of 1% w/v solution of carrageenan (Sigma Chemical Co, St. Louis, MO, USA) in to the plantar side of the right hind paw. The paw was marked with ink at the level of lateral malleolus and immersed in mercury up to the mark. A plethysmometer was used for the measurement of rat paw edema volume using the method reported by Singh and Ghosh[7]. The paw volume was measured immediately after injection (0 h) and then every hour till 3 h after injection of carrageenan to each group. The difference between the initial and subsequent reading gave the actual edema volume. The average paw swelling in the group of the drug treated rats was compared with untreated control rats and the percent of inhibition of the edema formation was determined using formula[8]: % inhibition = 100x(1-Vt/Vc), where ‘Vc’ represents edema volume in control and ‘Vt’ edema volume in the group treated with test drug. The results represented as % inhibition of inflammation are presented in Table 1. The values are mean±SEM (standard error of mean) of five animals in each group, statistically significant from control, p<0.001% inhibition given in parenthesis. The statistical analysis was carried by ANOVA followed by Student's t test.

**TABLE 1:** ANTIINFLAMMATORY ACTIVITY OF *PIPER LONGUM* DRIED FRUIT'S OIL

Group	Edema volume (ml)* at time (h)			Percent Inhibition of inflammation at time (h)		
	
	1	2	3	1	2	3
Control	0.33±0.12	0.59±0.26	0.94±0.47	-	-	-
*Piper longum* oil 0.5 (ml/kg)	0.17±0.08	0.27±0.12	0.32±0.20	48.48	54.23	65.95
1.0 (ml/kg)	0.12±0.09	0.20±0.11	0.26±0.08	63.63	66.10	72.34
Ibuprofen 100 (mg/kg)	0.11±0.06	0.19±0.08	0.28±0.097	65.62	67.79	70.21

* Values are mean±SEM, n=5 in each group. p<0.001 as compared to control group.

In the Indian system of medicine certain herbs are claimed to provide relief of pain and inflammation. In the present work, one such drug *Piper longum* dried fruit's oil was taken for the study. The results obtained indicate that the *Piper longum* oil had significant antiinflammatory activity in rats as shown in Table 1. This antiinflammatory activity was dose dependant and found to be statistically significant at the higher concentration, 1 ml/kg. The antiinflammatory activity of ibuprofen; a standard reference drug, was also found to be significant. This activity appears to be significant as carrageenan induced paw edema was taken as prototype of exudative phase of inflammation, where development of edema being described as biphasic[9]. The initial phase is attributable to release of various biochemicals, viz. histamine, 5-HT, various kinins in the first hour injection of carrageenan. A more pronounced second phase is related to the release of prostaglandin like substances in 2 to 3 h[10,11]. The essential oil of *Piper longum* reduced the edema induced by carrageenan by 65.95% on oral administration of 0.5 ml/kg and 72.34% on oral administration of 1 ml/kg, as compared to the untreated control group. Ibuprofen at 100 mg/kg inhibited the edema volume by 70.21%. Thus it can be concluded that the antiinflammatory activity of *Piper longum* oil is more potent at 1 ml/kg than at 0.5 ml/kg. The most remarkable point of this study was that the oil of *Piper longum* at 1 ml/kg produced more inhibition of edema than the standard antiinflammatory drug, ibuprofen.
